# Combined effect of mineralogical and chemical parameters on swelling behaviour of expansive soils

**DOI:** 10.1038/s41598-021-95746-5

**Published:** 2021-08-16

**Authors:** Bendadi Hanumantha Rao, Peddireddy Sreekanth Reddy, Bijayananda Mohanty, Krishna R. Reddy

**Affiliations:** 1 School of Infrastructure, IIT Bhubaneswar, Khordha, Odisha 752050 India; 2 Department of Civil Engineering, NIT Mizoram, Aizawl, 796012 India; 3 Department of Civil, Materials, and Environmental Engineering, University of Illinois at Chicago, Chicago, USA

**Keywords:** Civil engineering, Geochemistry

## Abstract

Microlevel properties such as mineralogical and chemical compositions greatly control the macro behaviour of expansive soils. In this paper, the combined effect of mineral (i.e. montmorillonite, MMC) and chemical contents (i.e. Ca and Na in their total (T), leachable (L) and exchangeable form (CEC)) on swelling behaviour is investigated in a comprehensive way. Several 3-dimensional (3D) graphs correlating MMC and Ca/Na ratio, together, with swelling property (swelling potential, *S*_*a*_, and swelling pressure, *S*_*p*_) are developed. 3D plots, in general, portrayed a non-linear relationship of *S*_*a*_ and *S*_*p*_ with MMC and Ca/Na ratio, together. It is hypothesized that swelling initially is triggered by chemical parameters due to their quick and rapid ionization capability, but the overall swelling phenomenon is largely controlled by MMC. It is importantly found that expansive soils are dominant with divalent Ca^++^ ions up to MMC of 67% and beyond this percentage, monovalent Na^+^ ions are prevalent. From the interpretation of results, the maximum *S*_*a*_ of 18% and *S*_*p*_ of 93 kPa is measured at MMC of 43%, (Ca/Na)_T_ of 10–14 and (Ca/Na)_L_ of 2–7. It is concluded from study that total CEC + MMC for determining *S*_*a*_ and (Ca/Na)_T_ + MMC for determining *S*_*p*_ are superior parameters to be considered. The findings of the study also excellently endorsed the results of Foster^32^, who stated that ionization of Na or Ca depends on the constituent mineral contents. The findings presented herein are unique, interesting and bear very practical significance, as no earlier research work reported such findings by accounting for chemical and mineralogical parameters impact, in tandem, on swelling properties.

## Introduction

Accurate prediction of expansive soils behaviour when hydrated with water is yet a major challenge faced by geotechnical engineers for long time. Expansive clays undergo volumetric deformations (viz., swelling and shrinkage) which in turn induce differential settlements leading to failure of a structure built in/on them or when they are used as geomaterials for construction purposes^1–7^. Numerous studies focused on understanding and evaluating the swelling behaviour by establishing interrelations with properties of physical, chemical and mineralogical^8–11^. However, most of the relationships so far developed are only 2-dimensional as they considered alone of physical or chemical or mineralogical features.

Demonstrably, swelling is the combined effect of chemical and mineralogical properties of a clay^12^. This statement, in general, is valid true for all natural soils, but prominently key for expansive soils because of the presence of montmorillonite mineral, which documented to be a principal cause for aberrant behaviour such as volume change characteristic. Minerals such as montmorillonite, kaolinite and illite are major compositions of clay soils. Lambe and Whitman^13^ documented the influence of different minerals on consistency limits in the order of: montmorillonite > attapulgite > illite > kaolinite. Generally, bentonite soils are rich in montmorillonite mineral, which is normally absent in natural soils^12,14^. The montmorillonite mineral upon imbibing of water, usually in significant quantity because of its remarkable surface area, tends to separate the clay sheets resulting in the formation of thicker diffuse double layer. Whereas in case of kaolinite, the absorption of water is limited to surface only, and thereby, effect is confined to marginal to nil.

Quantification of chemical and mineralogical parameters and relating their combined effect bears a practical significance for accurate prediction and precise determination of swelling behaviour. The quantification of chemical compositions, which would be achieved with the help of X-ray florescence (XRF) and inductively coupled plasma-optical emission spectrometry (ICP-OES) techniques, is relatively easy. However, the mineralogical quantification is a complex process and requires highly sophisticated equipment such as X-ray Diffraction (XRD), which is a commonly employed technique for establishing the diffraction patterns of soils. By giving the obtained diffraction pattern as input data, mineral content can be quantified using software’s like Topas. Methodologies such as External Standard Method^15^, Reference Intensity Ratio (RIR)^16^, Rietveld Method^17^, No-standard Method^18^, Mineral Intensity Factor (MIF)^19^, and Full Pattern Summation Methods^20^ are widely used for such purpose. Among the above methods, Rietveld method is reported to be the most reliable method for quantifying the minerals content^21^.

Several studies reported that the montmorillonite content is the prime factor behind the higher consistency and swelling behaviour of expansive soils^22–27^. In connection to this, Sun et al.^28^ predicted the swelling pressure of bentonite and sand mixes by developing empirical relationship between void ratio and swelling pressure. In similar lines, Chittoori et al.^29^ studied the effect of clay mineral montmorillonite on the performance of chemically stabilized soils, and Mehta and Sachan^30^ investigated the influence of mineralogy on mechanical behaviour of expansive soils. More deeply, Tahasildar et al.^11^ studied the influence of montmorillonite on swelling behaviour with limited set of data. Recently, Reddy et al.^31^ quantified the montmorillonite content and established relationships with swelling behaviour determined by free swell index test.

Besides the noteworthy influence of mineralogy, the effect of chemical parameters on swelling behaviour cannot be simply ignored. Foster^32^ reported that the soils dominated with Na tends to swell more than those Ca does. As per Chen^12^, soils dominated with sodium (Na) and calcium (Ca) can greatly affect the swelling behaviour of expansive soils. This occurs as these chemicals exhibit affinity to adsorb substantial amount of water that in turn inflates the thickness of diffuse double layer^33,34^. Many studies also reported that Na and Ca contents could stimulus the swelling behaviour^35–37^. Shainberg and Letey^38^ stated that, higher is the concentration of monovalent cations (i.e. Na), greater is the swelling in soils. Most of the literature, however, confined to highlighting a fact that there is an influence of chemical parameters on swelling, but very a few efforts are oriented to quantify and establish generalized relationships.

From the foregoing discussion, it is evident that both mineralogical and chemical parameters have a combined impact on the swelling behaviour. But studies pivoted on analyzing the combined effect of these parameters, which are crucial factors controlling the macro behaviour such as volume change of clayey soils, are teensy. The main objective of this paper is to understand the combined impact of mineralogical and chemical parameters on the swelling behaviour. For experimentation purpose, several expansive soil samples were collected from different locations across India. Sodium (Na), calcium (Ca) and montmorillonite content (MMC) of these soil samples were quantified. For vivid understanding, 3-D graphs relating chemical and mineralogical parameters with swelling parameters were developed. All total, leachable and exchangeable forms of Na and Ca were considered for developing the 3D graphs. Evidently, such combined analysis is indispensable for accurately determining the swelling characteristics as well as to validate the empirical equations proposed by various researchers. The results presented in the study can also serve as guiding factors for practical applications to assess swelling potential based on input of Ca/Na ratio and MMC values, to decide upon whether or not to go for construction on expansive soils, and importantly, as a tool to practical engineers for the selection of proper remedial measure which is more compatible to the media with which it is implemented. Overall, the study portrays that it is important to merge the effect of chemical and mineralogical parameters for reliable understanding on the swelling behaviour of expansive soils.

## Materials and test methodology

**Figure 1:**
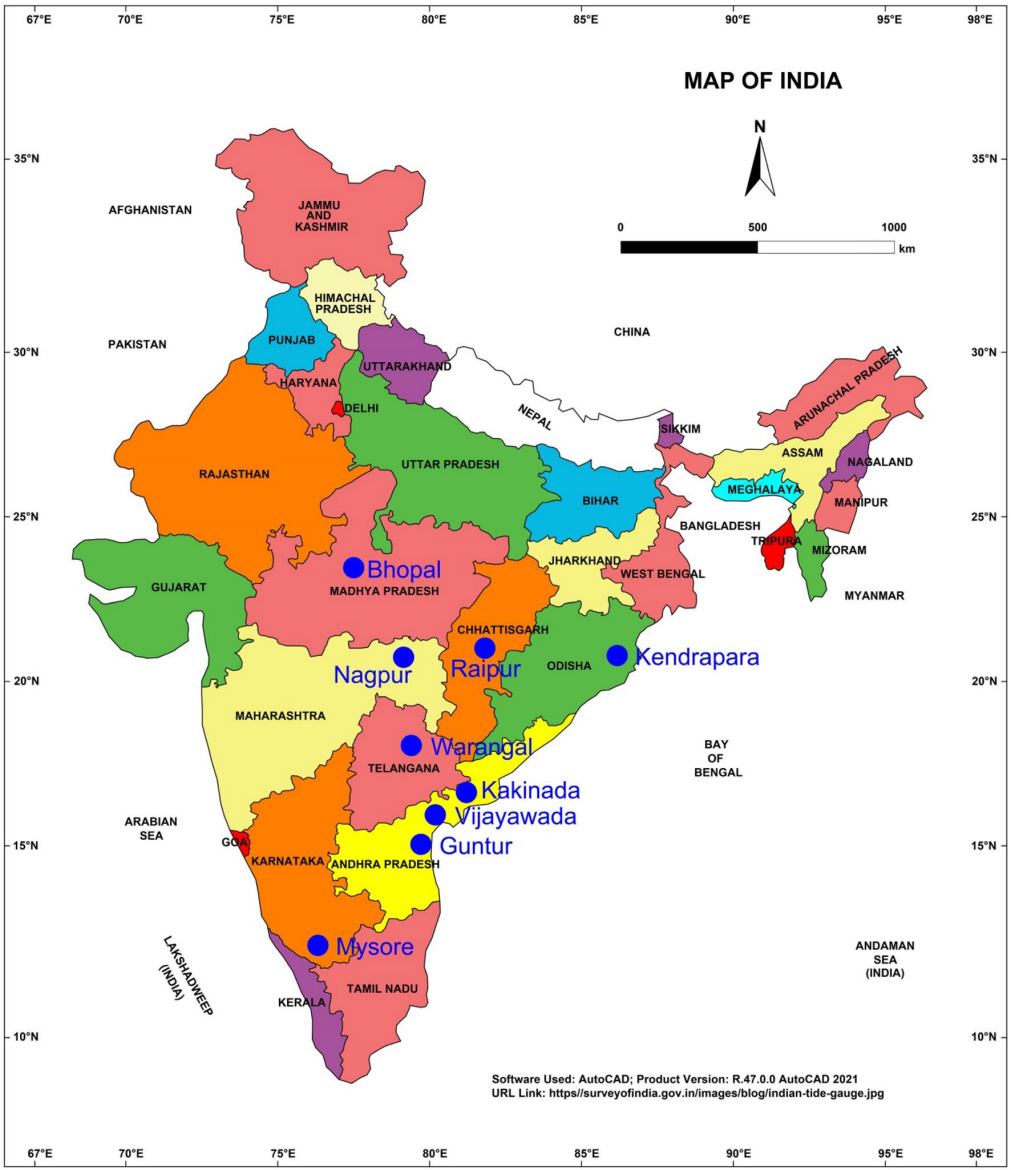
Geographical map of India showing regions from where soil samples were collected for the study purpose.

For the study purpose, total 46 soil samples of expansive in nature from nine different locations across India were collected following the guidelines of ASTM D1452^39^, and were used. Figure 1 depicts the geographic status showing the locations of soil samples from where they were collected. Whereas, Table 1 presents the latitude and longitude of the exact location of samples collection. All the soil samples were primarily observed to be blackish grey color and they were generally termed as “black cotton soils” in Indian context^40^. Soil environment of all the regions was found predominantly semi-arid type, which experiences climates of hot during the summer and precipitation during the rainy season. As regards temperature, the minimum and maximum was recorded in the range of 10.2–19.2 °C and 38–48.5 °C^41–43^, and that of humidity in the range of 21–84%^41–43^. These regions were reported to be receiving an annual average rainfall from 776.7 to 1637 mm. The geological formations of these regions include granite, gneiss, schist, basalt, and sandstone. A typical chemical weathering of the rocks dominated with fines content seems to be a fundamental cause for soil formations in these regions.

**Table 1:** Latitude and longitude of soil samples collection region and their designation.

Sl. No	Sample Region	Sample Designation	Latitude	Longitude
1	﻿Bhopal	B1	23° 13′ 01.35″	77° 24′ 13.52″
		B2	23° 12′ 20.90″	77° 26′ 44.89″
		B3	23° 14′ 46.23″	77° 30′ 39.32″
		B4	23° 14′ 51.10″	77° 30′ 36.68″
		B5	23° 14′ 55.33″	77° 15′ 45.95″
		B6	23° 18′ 23.81″	77° 19′ 56.12″
		B7	23° 19′ 46.14″	77° 24′ 09.19″
		B8	23° 06′ 33.45″	77° 30′ 31.85″
2	Guntur	G1	16° 16′ 25.29″	80° 28′ 15.07″
		G2	16° 16′ 25.29″	80° 28′ 15.07″
		G3	16° 18′ 17.10″	80° 28′ 58.38″
		G4	16° 18′ 37.48″	80° 28′ 27.75″
		G5	16° 19′ 09.82″	80° 27′ 01.86″
		G6	16° 19′ 42.28″	80° 24′ 23.03″
		G7	16° 19′ 12.30″	80° 23′ 41.06″
3	Kakinada	K1	16° 58′ 53.74″	82° 12′ 59.65″
		K2	16° 57′ 38.28″	82° 15′ 42.06″
		K3	17° 00′ 36.79″	82° 13′ 31.09″
4	Nagpur	N1	21° 03′ 39.82″	79° 04′ 24.52″
		N2	21° 03′ 54.54″	79° 07′ 33.57″
		N3	21° 03′ 55.79″	78° 57′ 24.19″
		N4	21° 08′ 44.04″	78° 59′ 12.58″
		N5	21° 11′ 02.49″	79° 02′ 01.82″
		N6	21° 15′ 31.20″	79° 05′ 11.50″
		N7	21° 11′ 38.33″	79° 07′ 51.29″
		N8	21° 08′ 33.62″	79° 10′ 59.73″
5	Raipur	R1	21° 12′ 01.53″	81° 38′ 43.15″
		R2	21° 12′ 20.00″	81° 36′ 58.98″
		R3	21° 18′ 19.52″	81° 41′ 38.72″
6	Vijayawada	V1	16° 28′ 45.92″	80° 41′ 43.00″
		V2	16° 30′ 00.49″	80° 42′ 14.07″
		V3	16° 31′ 40.29″	80° 40′ 51.18″
		V4	16° 33′ 11.00″	80° 39′ 43.29″
		V5	16° 32′ 59.68″	80° 38′ 00.92″
		V6	16° 32′ 53.56″	80° 36′ 23.34″
		V7	16° 28′ 48.00″	80° 36′ 24.50″
7	Warangal	W1	17° 58′ 07.12″	79° 30′ 22.63″
		W2	17° 57′ 05.08″	79° 35′ 09.70″
		W3	17° 59′ 57.95″	79° 36′ 26.24″
8	Mysore	M1	12° 16′ 32.20″	76° 39′ 29.08″
9	Kendrapara	KP1	20° 41′ 27.95″	86° 09′ 38.17″
		KP2	20° 40′ 17.68″	86° 10′ 08.10″
		KP3	20° 35′ 26.50″	86° 16′ 52.63″
		KP4	20° 34′ 33.39″	86° 17′ 36.67″
		KP5	20° 30′ 36.43″	86° 22′ 45.35″
		KP6	20° 25′ 24.66″	86° 29′ 47.82″

The samples collected were dried and pulverized as per the test standard ASTM D2216^44^. The processed samples were later subjected to extensive experimental investigations, including particle size distribution^45^, consistency limits^46^, swelling properties, mineralogical and chemical compositions. As per the Unified Soil Classification System (USCS)^47^, the soils were classified as Inorganic clays of high compressibility (CH) based on gradation and index properties. The physical properties of soil samples used in the study can be seen in Table 2.

**Table 2:** Physical properties of soil samples used in the study.

State/Region	Sample ID	% Fraction				Consistency limits (%)			USCS
		Gravel (> 4.75 mm)	Sand (4.75 mm–75 µm)	Silt (75 µm–2 µm)	Clay (< 2 µm)	*w*_*L*_	*w*_*P*_	*w*_*PI*_	
Bhopal	B1	–	6	43	51	68	27.52	40.48	CH
	B2	–	6	50	44	57.5	23.54	33.96	CH
	B3	–	3	51	46	62	27	35	CH
	B4	–	7	53	40	63.9	21.42	42.48	CH
	B5	–	17	44	39	43	19.25	23.75	CL
	B6	1	23	43	33	48	18.52	29.48	CL
	B7	1	21	42	36	46	16.9	29.1	CL
	B8	1	17	40	42	50.8	23.5	27.3	CL
Guntur	G1	–	11	31	58	93	36	57	CH
	G2	–	26	37	37	78	25.78	52.22	CH
	G3	1	40	29	30	43	19.35	23.65	CL
	G4	–	8	47	45	68	24	44	CH
	G5	–	32	32	36	44.83	21.23	23.6	CL
	G6	–	10	33	57	80	29.53	50.47	CH
	G7	–	13	28	59	88	27.63	60.37	CH
Kakinada	K1	–	5	46	49	82	31	51	CH
	K2	–	3	42	55	73.6	29.78	43.82	CH
	K3	–	4	40	56	70.2	27.65	42.55	CH
Nagpur	N1	2	14	43	41	61	21.92	39.08	CH
	N2	4	16	38	42	58	23	35	CH
	N3	3	26	38	33	48	20.85	27.15	CL
	N4	1	22	30	53	57.7	22.52	35.18	CH
	N5	3	19	29	51	69	24.68	44.32	CH
	N6	2	21	28	49	61.9	29.76	32.14	CH
	N7	1	14	32	53	74	27.24	46.76	CH
	N8	3	13	30	54	66	27.16	38.84	CH
Raipur	R1	1	15	39	45	76.7	25.39	51.31	CH
	R2	0	10	43	47	62	23	39	CH
	R3	0	14	38	48	57	25.54	31.46	CH
Vijayawada	V1	–	12	33	55	92	34.32	57.68	CH
	V2	2	18	35	45	43	22	21	CL
	V3	2	20	32	46	46	17.7	28.3	CL
	V4	3	22	33	42	46.1	18.93	27.17	CL
	V5	1	17	39	43	52	15.54	36.46	CH
	V6	–	9	40	47	82.8	26.43	56.37	CH
	V7	–	11	39	50	79.8	26.20	53.6	CH
Warangal	W1	2	26	30	42	70	26	44	CH
	W2	–	27	29	44	51.5	23.27	28.23	CH
	W3	1	43	28	28	53.45	22.89	30.56	CH
Mysore	M1	–	21	26	53	72	28.35	43.65	CH
Kendrapara	KP1	4	15	38	43	60	28.25	31.75	CH
	KP2	3	17	33	47	53	29.54	23.46	CH
	KP3	2	18	29	51	70	32	38	CH
	KP4	3	21	31	45	42.5	19.5	23	CH
	KP5	4	20	29	47	51	23.73	27.27	CH
	KP6	2	19	35	44	68	22.87	45.13	CH

### Swelling characteristics

Swelling characteristics (i.e. *S*_*a*_ and *S*_*p*_) of each soil sample were measured in accordance with the guidelines of ASTM D2435^48^ standard. Samples of oven-dried and passing 425 microns sieve were used for the preparation of test specimen. Swelling tests were carried out with the help of a conventional oedometer apparatus, which houses an oedometer ring of 60 mm internal diameter and 20 mm height for accommodating the specimen. Each soil sample was compacted to maximum dry density at optimum moisture content inside the ring up to the height of 12 mm. Three LVDTs (with least count of 0.001 mm) to measure the change in height (i.e. deformation) of specimen with time in three different directions were mounted and an average of them was considered representative value. The test was commenced by flooding the sample with water, supplied from an overhead reservoir, and it is continued until the time taken to attain three consecutive values of deformations were identical. The final deformation when divided with initial height yields the swelling potential, *S*_*a*_, of a soil sample. From the stage of final deformation, the sample was compressed by gradually applying compressive pressure with initially from zero and in steps of 10 kPa until the deformation reaches to 0 or negative^49^. The pressure corresponding to zero deformation was determined, which yields the swelling pressure, *S*_*p*_.

### Quantification of mineral and chemical parameters

The objective of the study is to investigate the combined effect of mineral (i.e. montmorillonite) and chemical parameters (sodium and calcium contents) on swelling property. Accordingly, efforts were devoted to quantify, first, these parameters in 46 numbers of soil samples. However, chemical parameters in soils can present in different forms such as total (designated as Na_T_ for sodium and Ca_T_ for calcium), leachable (designated as Na_L_ for sodium and Ca_L_ for calcium), and exchangeable (designated as Na_CEC_ for sodium and Ca_CEC_ for calcium)^50^, in disproportionate quantity. The total form is the direct image of structural chemical element of a given soil sample^51^, whereas the leachable form is the quantity of structural chemical element that leaches out under the influence of concentric acids^52^. On the other hand, exchangeable form is the quantity of cations hold by clay particles and are ready to exchange with other cations when fluid comes in contact^53^.

XRF and ICP-OES are widely used analytical techniques to determine elemental compositions of soil samples. XRF is a rapid technique for analyzing the total form of an element (i.e. Na_T_ and Ca_T_) in dry soil samples accurately. Whereas, ICP-OES technique is basically meant for analyzing aqueous samples to determine major and trace elements in leachable form (i.e. Na_L_ and Ca_L_), specifically in natural soils, polluted soils, and biological samples. These chemical parameters, prima facie, represent mean structural chemical parameters of different mineral components. However, the swelling is attributed the exchange of cations (i.e. cation exchange capacity, CEC), which broadly manifests external chemical environment. Therefore, besides the quantification of Na and Ca contents in their total and leachable form, the exchangeable form of these individual elements (i.e. Na_CEC_, Ca_CEC_), their ratio (i.e. (Ca/Na)_CEC_) and combination (i.e. total CEC) were also picked up for the study purpose. In this regard, an additional data pertinent to exchangeable form of Ca, Na and total contents for expansive soils were collected from the literature.

Holistically, it is incorrect to consider the effect of individual element/ion. Therefore, their ratio (i.e. (Ca/Na)_T_, (Ca/Na)_L_, and (Ca/Na)_CEC_) was chosen as studying chemical parameters to derive a vivid understanding of them along with MMC.

### Mineralogical characteristics

#### Separation of clay size particles

Orientation of clay particles has a dominant impact on quantification of clay minerals^54,55^. Errors in the quantification of clay minerals can be pronounced if there are no preferred orientations, which can severely be disturbed in the presence of non-clay fine particles^54^. In order to increase the rate of accuracy in quantification, the planes (001) should be oriented parallel to the surface^56^. For achieving this, the clay fraction in the soil sample needs to be segregated. To accomplish this, wet sieve analysis was performed using sieve with aperture size of 20 microns. The procedure postulated by Brandt et al.^57^ was followed for this purpose. The suspension derived was collected in a container and was oven dried at 105 ± 0.5 °C for 24 h. Specimens obtained subsequent to drying were pulverized to down size the particles with the help of masher. 2 to 3 g of fine soil was grabbed from this pulverized fraction and was transferred into the centrifuge tube after adding 1% calgon solution. The mixture was ultra-sonicated for 1 min to disperse the particles prior to the centrifugation. The suspension was centrifuged at a speed of 1000 rpm for 5 min, so that larger particles settle at the bottom of the tube. After centrifugation, calgon solution was decanted into another centrifuge tube and the same was centrifuged again at 1500 rpm for 15 min. After decanting the calgon solution again, the residue deposited at the bottom of the tube was washed with distilled water. The above steps of decantation and centrifugation were repeated for 3 to 5 times. Finally, 2 to 3 drops of the suspension were placed on a glass slide by uniformly spreading with glass rod and allowed to dry overnight at temperature of 60–70 °C in the desiccator containing ethyl glycol solution, which was already filled to 1 cm deep from the bottom. Ethylene glycol expands the clay minerals such as montmorillonite, smectite, and other mixed minerals for easy identification and their quantification^57^. Another advantage of this treatment is it causes fewer disturbances in orientations along with the amorphous scattering of X-rays by excess liquid than other methods^57^. Then the dried sample over glass slide is fed to the equipment for further analysis.

#### Quantification of montmorillonite content

The mineralogical composition of a soil was determined with the help of D8 Advanced X-ray powder diffraction device (make, BRUKER, USA). The sample from the desiccator was directly mounted on the equipment platform and scanned for reflections with a voltage and current of 40 kV and 40 mA, respectively, with 2θ ranging from 5 to 80° with a step size of 0.025° and time interval of 0.5 s for each step using a copper X-ray tube (i.e. Cu-Kα radiation). X-ray beams were directed to hit the sample such that process scatters the atoms in their path. Post scattering, structural characteristics of the sample were detected by applying Bragg’s law^58^. The presence of minerals in each sample was identified with the help of DIFFRAC.SUITE EVA V.3.0^58^ software. The software performs automatic search on raw data, which was background subtracted automatically, and on the peak list to identify the most appropriate mineral phase.

**Figure 2:**
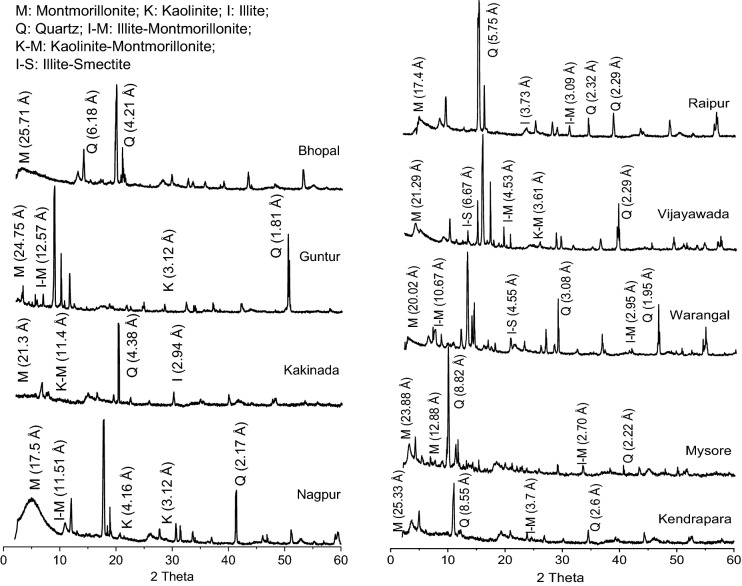
X-ray diffraction pattern of a soil sample designated with dominant minerals identified.

Montmorillonite content, MMC, in each soil sample was quantified using TOPAS 4.2 software, which takes whole pattern as input data. Different minerals in each soil sample was identified by comparing the obtained diffraction data with the International Centre for Diffraction Data base (ICDD). Once the phases have been identified, the necessary atomic information was extracted from the database to produce a computed profile. However, quantitative clay mineralogy requires mineral standards with XRD properties similar to those of mineral phases in unknown samples^59^. For this purpose, Inorganic Crystal Structure Database (ICSD) reference patterns were employed to match with the measured pattern. TOPAS 4.2^60^ is a graphics based profile analysis program and it integrates various types of X-ray and neutron diffraction analyses by supporting all profile fit methods currently employed in powder diffractometry. The software by resorting to the Rietveld technique computed the mineralogical quantitative analysis^61^. For the sake of clarity, diffraction pattern established on soil sample from each region is presented in Fig. 2.

Rietveld refinement performs on whole pattern basis which is a powerful approach for quantifying the identified minerals. In this method, the reflections are replicated with the help of calibrated crystallographic parameters^17,62–64^. Whereas in the case of single reflection method, individual peak is considered for the quantification of clay minerals, which requires mineral standards^21^. Generally, there are two types of internal standards such as ZnO (Zincite) and α-Al_2_O_3_ (corundum) for quantifying the minerals in clay based soil samples. The former and latter standards are normally used in Mineral Intensity Factor^65^ and Reference Intensity Ratio methods^19^. Conforming this fact, Srodon et al.^65^ and Hillier^66^ employed Zincite and Corundum as internal standards for quantifying the minerals. The software performs the least square method for quantitative phase analysis on the data extracted and known data from ICSD. The difference between the observed and known structural factors such as site occupancy information, cell dimension, inter-atomic distance, temperature, and magnetic factors was minimized by multiple iterations. Later on successful completion of scale factor (i.e. atomic positions, thermal vibrations), different quantities of minerals were obtained^67^.

#### Quantification of Na_T_ and Ca_T_ by X-ray fluorescence spectroscopy (XRF)

Total form of sodium and calcium contents in each soil sample was determined with the help of X-ray fluorescence device (make, PANalytical, The Netherland). The soil sample used for analysis purpose was in pellet form. The palletization was accomplished by filling 32 mm diameter aluminium cup with boric acid powder at the bottom first and then with dry soil sample. The boric acid is selected because it doesn’t consist of trace elements. Approximately 5–8 g of oven-dry soil passing sieve size of 75 μm was used. The cup filled up with boric acid and soil sample was transferred to automatic press apparatus and a load of 20 ton was applied for the duration of 20 s. This process transforms the powder sample into pellet, which was then utilized for XRF analysis to measure calcium and sodium contents. Each sample was scanned for reflections with a voltage and current of 60 kV and 50 mA, respectively, using a copper X-ray tube (i.e. Cu-Kα radiation). Soil sample when exposed to X-ray source, elements in the sample produce a specific sign/emission, which is distinct for every element. By counting the intensity of sign and wavelength, quantification of different elements shall be made^68^. The analysis quantifies range of each element accurately from 0.01–100% of total content by its dry weight basis^69^. The results in elemental form were then converted into their oxides form of soils^70^ .

#### Quantification of Na_L_ and Ca_L_ by inductively coupled plasma-optical emission spectrometry (ICP-OES)

ICP analysis gives leachable form of chemical elements and it is generally performed on solutions derived by digesting soil samples with high concentrated acids, as per the guidelines of USEPA 3050B^71^ standard. The digestion involves addition of 0.5 g of soil to 9 ml of HNO_3_, 3 ml of HF, 2 ml of HCl, and 5 ml of distilled water. In case of soil sample containing any organic matter, 2 ml of H_2_O_2_ was added additionally. The solution mix was then transferred to vessels made of Polytetrafluoroethylene (PTFE). The vessels capped to ensure air or water tightness were then placed inside the microwave digester and the soils mixtures were digested for the duration of 60 min. The resultant mixture was filtered through 42 micron Whatman filter paper to segregate liquor and residue. The filtrate solution thus obtained was fed to ICP-OES device (make, PerkinElmer, Model: Avio 200) for the analysis purpose. The liquid when exposed to plasma energy, constituent elements i.e. atoms, gets excited. The type of element was determined by employing plasma energy and its quantity is obtained from the intensity of photon rays. The chemical compositions determined from XRF and ICP analysis can be seen in Table 3.

**Table 3:** Chemical properties of soil samples used in the study.

State/Region	Sample ID	XRF Analysis		ICP Analysis	
		Ca_T_ (%)	Na_T_ (%)	Ca_L_ (mg/kg)	Na_L_ (mg/kg)
Bhopal	B1	2.602	0.37	63.71	75.57
	B2	10.303	0.402	100.5	135.3
	B3	3.03	0.42	61.29	88.9
	B4	4.081	0.518	74.31	160.6
	B5	3.886	0.417	82.47	104.3
	B6	5.065	0.38	84.29	125.3
	B7	10.515	0.382	79.8	97.3
	B8	4.201	0.298	70.6	129
Guntur	G1	5.796	0.517	76.9	108.4
	G2	8.675	0.811	123.1	200.4
	G3	5.032	0.719	98.25	207.9
	G4	3.442	0.258	73.22	81.72
	G5	5.253	0.634	68.08	222.9
	G6	4.51	0.427	62.13	224.5
	G7	2.5	0.314	110.5	123.6
Kakinada	K1	3.846	0.463	66.19	70.84
	K2	3.04	0.338	39.69	170.1
	K3	3.182	0.409	50.03	74.73
Nagpur	N1	6.858	0.334	89.94	48.26
	N2	5.229	0.401	77.95	67.17
	N3	3.502	0.287	55.71	108
	N4	5.25	0.213	96.85	57.5
	N5	3.792	0.228	69.74	129.9
	N6	5.369	0.267	62.28	47.18
	N7	5.252	0.231	60.68	47.07
	N8	4.636	0.225	114.1	52.38
Raipur	R1	4.271	0.181	46.94	53.4
	R2	4.286	0.249	43	41.02
	R3	2.059	0.154	25.32	112
Vijayawada	V1	7.067	0.626	185.3	164.4
	V2	5.532	0.891	149.4	189.8
	V3	4.713	0.753	96.6	148.8
	V4	5.494	0.738	63.02	105.8
	V5	4.476	0.681	68.31	197.5
	V6	4.271	0.677	103.7	136.5
	V7	4.746	0.725	74.38	99.82
Warangal	W1	3.906	1.12	63.17	103
	W2	3.301	0.648	50.98	106.7
	W3	3.859	1.405	71.95	277
Mysore	M1	2.599	0.349	101	143.5
Kendrapara	KP1	1.161	0.178	27.82	120.2
	KP2	1.56	0.427	36.44	120.7
	KP3	2.226	0.464	26.08	154.3
	KP4	1.327	0.293	21.91	75.8
	KP5	1.322	0.432	18.68	136.7
	KP6	1.294	0.435	26.71	132.1

## Results

**Figure 3:**
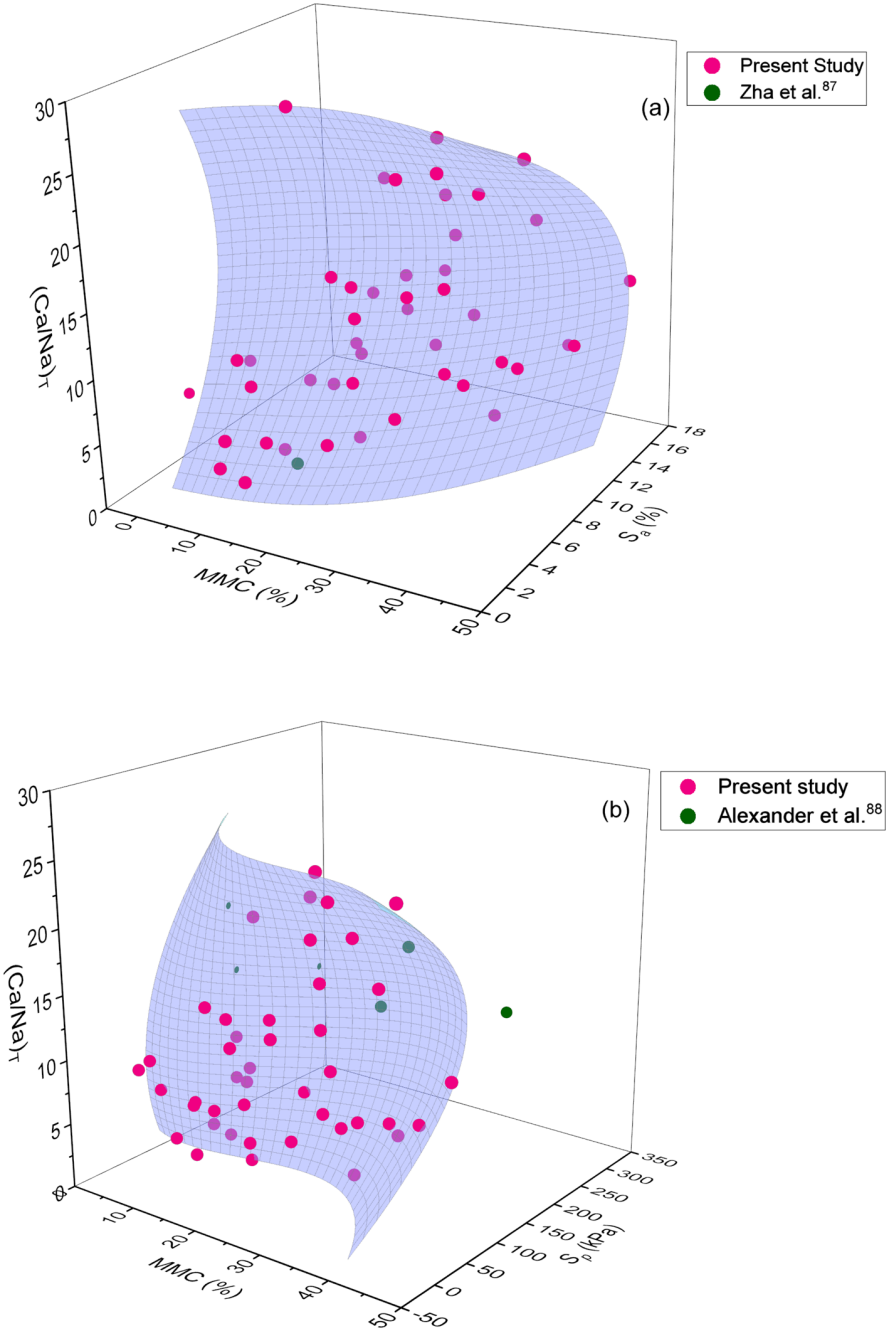
Combined influence of MMC and (Ca/Na)_T_ on (**a**) swelling potential and (**b**) swelling pressure of expansive soils.

**Figure 4:**
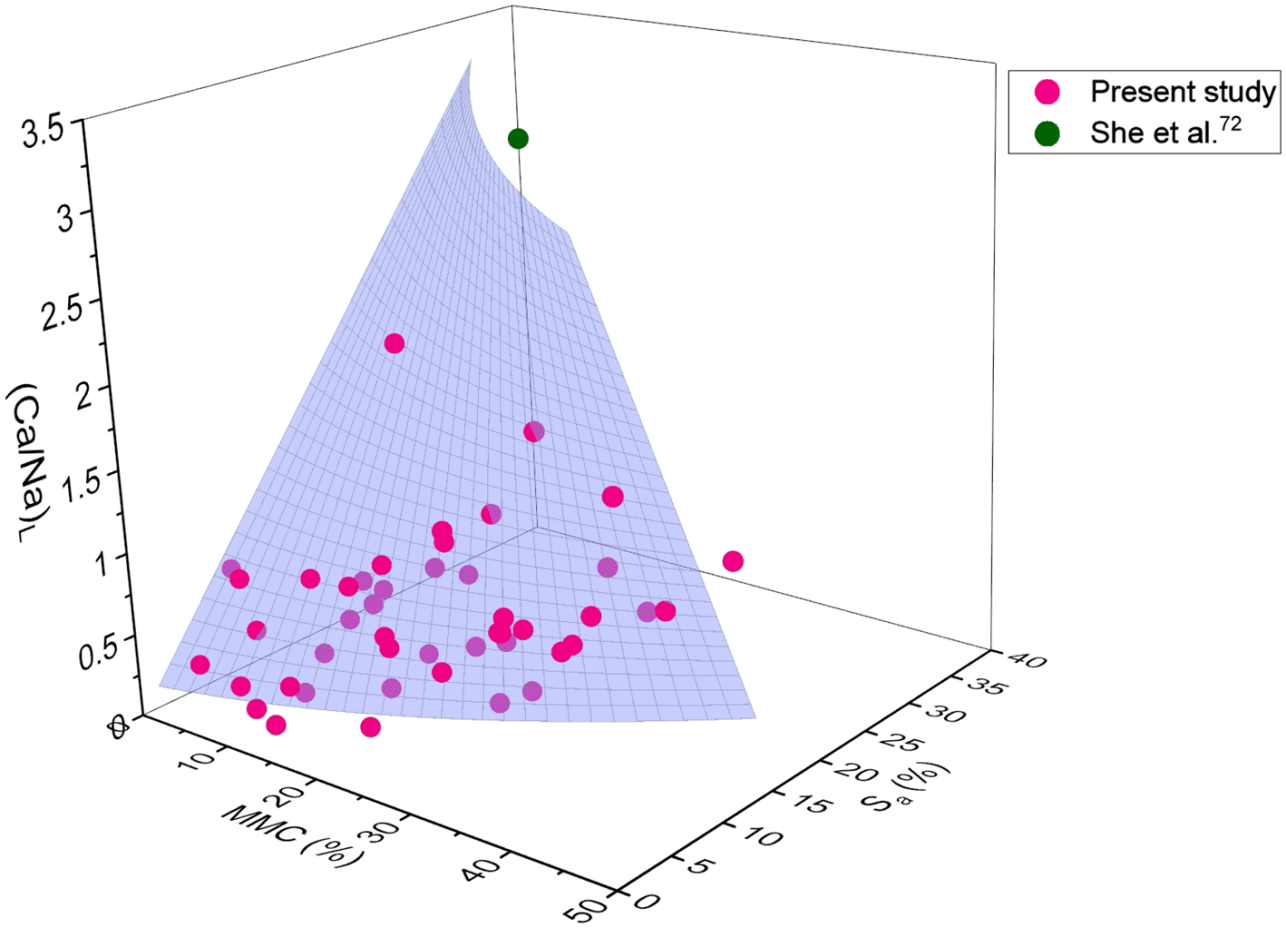
Combined influence of MMC and (Ca/Na)_L_ on swelling potential of expansive soils.

**Figure 5:**
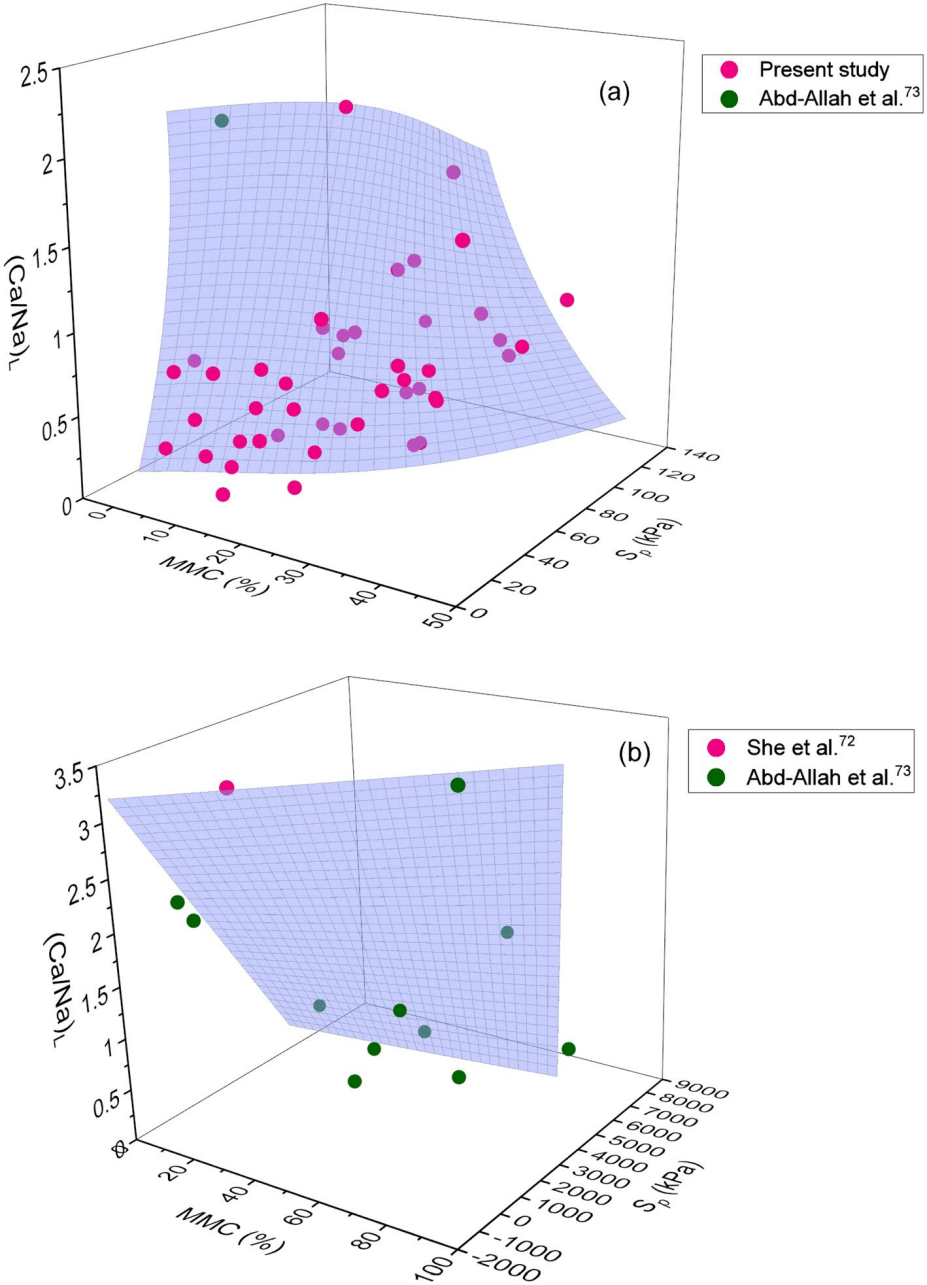
Combined influence of MMC and (Ca/Na)_L_on the swelling pressure of expansive soils (**a**) *S*_*p*_ < 120 kPa and (**b**) *S*_*p*_ > 120 kPa.

**Figure 6:**
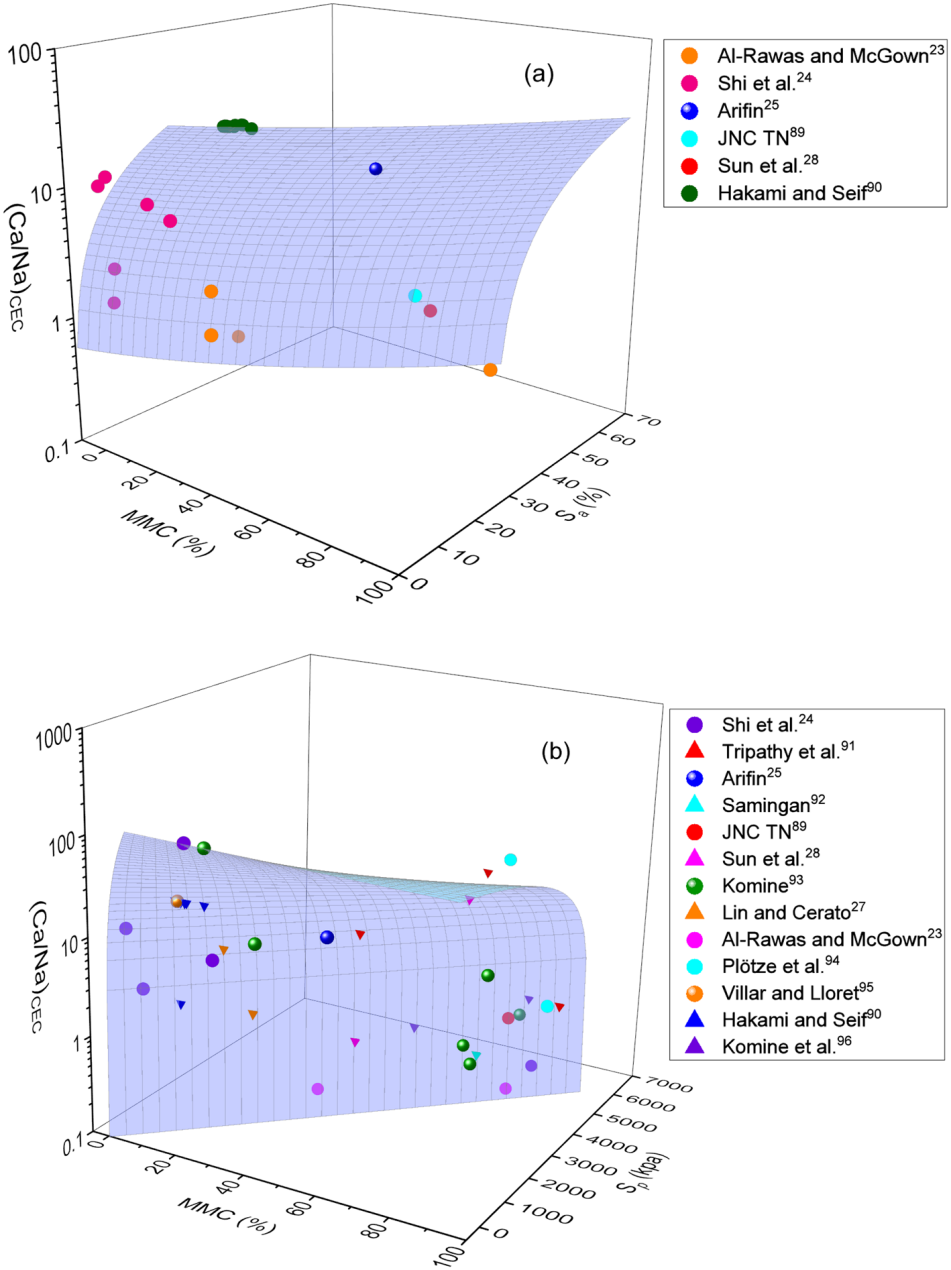
Combined influence of MMC and (Ca/Na)_CEC_ on (**a**) swelling potential and (**b**) swelling pressure of expansive soils.

**Figure 7:**
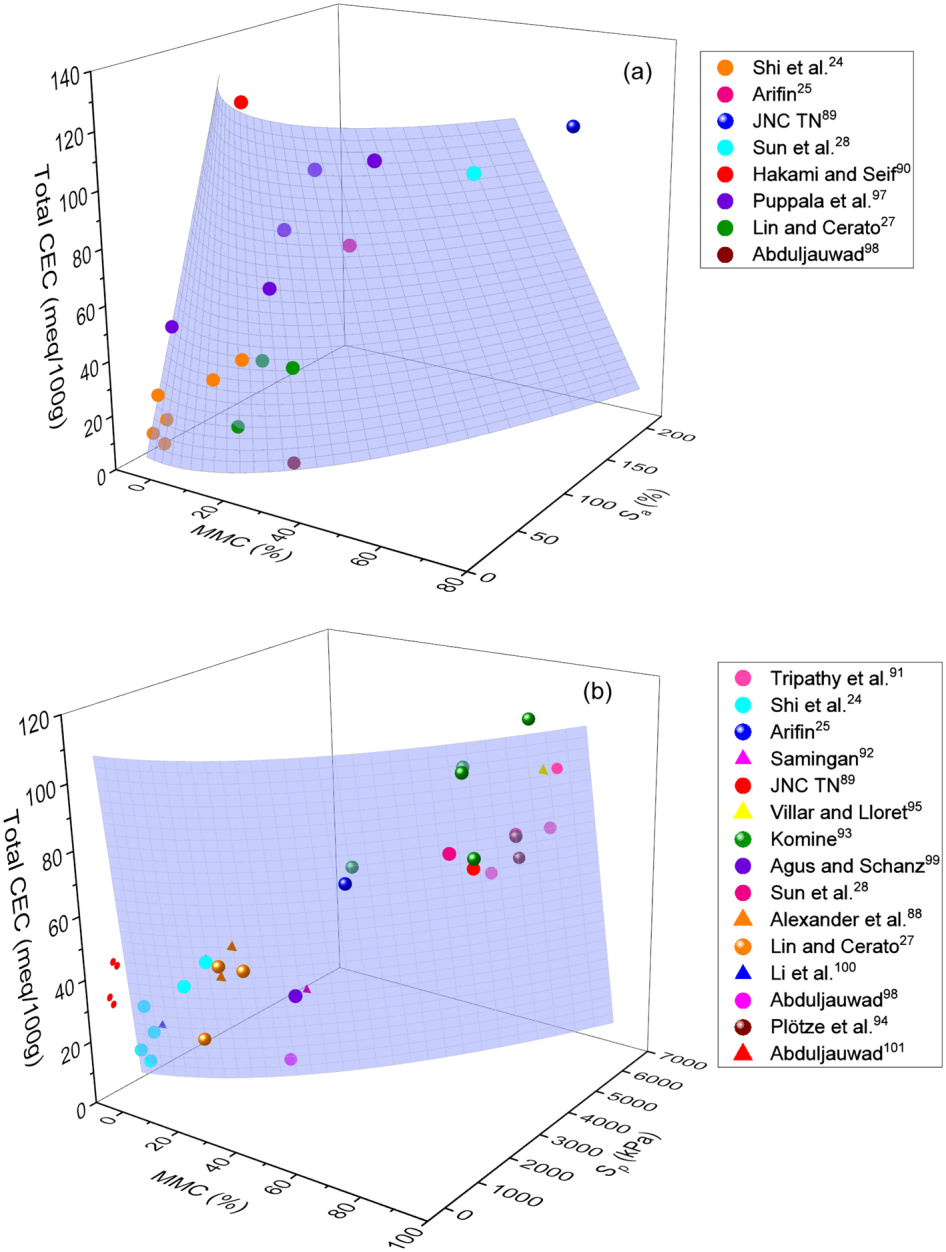
Combined influence of MMC and total CEC on (**a**) swelling potential and (**b**) swelling pressure of expansive soils.

Based on the results obtained from extensive experimentations, 3D graphs demonstrating the combined effect of chemical and mineralogical parameters on swelling parameters were developed, as depicted in Figs. 3, 4, 5, 6 and 7 respectively. Please note that chemical parameters effect includes separately for structural chemical parameters (i.e. (Ca/Na)_T_ and (Ca/Na)_L_) and external chemical environment (i.e. (Ca/Na)_CEC_). To delineate the exact impact, 3D polynomial surface curve fits relating (Ca/Na)_T/L/CEC_ and MMC with *S*_*a*_ and *S*_*p*_ were employed to the data, as shown in Figs. 3, 4, 5, 6 and 7.

A clear concave shape polynomial surface profile, which extends towards higher values of MMC, corresponding to *S*_*a*_ and *S*_*p*_ can be viewed from Fig. 3. It is obvious that the combined effect of Ca/Na ratio and MMC on *S*_*a*_ and *S*_*p*_ appears to be non-linear in nature. It is seen from Fig. 3 that with an increase in MMC and (Ca/Na)_T_, both *S*_*a*_ and *S*_*p*_ increased (diagonally upward), reached a peak and then receded. It can be noticed that when MMC and (Ca/Na)_T_ ratio is least that is near the origin of the graph, the values of *S*_*a*_ and *S*_*p*_ are also trivial. As evident from Fig. 3, the influence of MMC and (Ca/Na)_T_ on *S*_*a*_ is well defined for *S*_*a*_, as it is increased in proportion to increment of MMC and (Ca/Na)_T_ parameter, in comparison with *S*_*p*_. Understandably on the other hand, the ratio of (Ca/Na)_T_ seems increases with an increase in MMC. Similarly, Figs. 4 and 5 show the polynomial surface fits employed to correlate *S*_*a*_ and *S*_*p*_ with (Ca/Na)_L_ and MMC parameters, in tandem. Two distinct correlations for *S*_*p*_ < 120 kPa and *S*_*p*_ > 120 kPa were formulated depending upon the trend revelations when data is drawn for *S*_*p*_ against (Ca/Na)_L_ and MMC. Thus, for the sake of clarity, two independent plots were developed, as depicted in Fig. 5. Please note that data used in Fig. 5b is explicitly collected from the literature. From Figs. 4 and 5a, it can be observed that both *S*_*a*_ and *S*_*p*_ increased linearly with MMC and (Ca/Na)_L_. Evidently, the ratio of (Ca/Na)_L_ is mostly confined to unity, and when it is exceeded this value, *S*_*a*_ is found to be significantly ascended although MMC is minimal (Fig. 4). It is also seen from Fig. 5, a reversal in trend for *S*_*p*_ above and below 120 kPa.

When (Ca/Na)_T_ exceeded 20, surface profile is apparently seen sloping towards MMC (Fig. 3). Similar such observation of surface profile inclining towards MMC can be noticed from Figs. 4 and 5b. In fact, a complete dominance of MMC over (Ca/Na)_L_ can be seen from Fig. 5b. These statements substantiate a likely supremacy of MMC over chemical parameters (i.e. (Ca/Na)_T,L_) on *S*_*a*_ and *S*_*p*_. From these statements, it can also be inferred that initial swelling might be triggered by chemical parameters and the subsequent volume change seems completely controlled by MMC. This is an interesting and important finding by the study, as no earlier research work reported such findings with a bearing on chemical and mineralogical parameters together on swelling properties.

A close observation of Figs. 3 and 4 reveals a wide variation of total form of chemical data vis-à-vis that of leachable form, which remains largely confined to less than unity. Conversely, a noticeable difference in effect can be perceived between total and leachable forms from Figs. 3, 4 and 5. It is interesting to note their distinct impact on *S*_*a*_ and *S*_*p*_, although, total and leachable forms are inherent constituents of a given soil. Contrary to the total form response that showed non-linearity with swelling parameters, leachable form depicts linear variation with *S*_*a*_ and *S*_*p*_. Furthermore, the influence of leachable form is remained same on both *S*_*a*_ and *S*_*p*_ up to 120 kPa, as profile curve fits have closely resembled each other between Figs. 4 and 5a. Based on the pronounced and marked influence of total form chemical parameters, it can be interpreted from Figs. 3 and 4 that resorting to total form is beneficial for precise determination of swelling behaviour, over leachable form. From Figs. 3, 4, and 5, the maximum *S*_*a*_ of 18% and *S*_*p*_ of 93 kPa is measured at MMC of 43%, (Ca/Na)_T_ of 10–14, and (Ca/Na)_L_ of 2–7. The results obtained in the present study are in well agreement with the studies reported by Shi et al.^24^, She et al.^72^, and Abd-Allah et al.^73^, who have reported properties of MMC, chemical and swelling parameters that are found to be falling in the range of data produced by the presented study.

Figures 6 and 7 show the variations of *S*_*a*_ and *S*_*p*_ with (Ca/Na)_CEC_, total CEC, and MMC parameters. Obviously, an increase in *S*_*a*_ and *S*_*p*_ with an increase in MMC, (Ca/Na)_CEC_, and total CEC, together, as well as a decrease and an increase in (Ca/Na)_CEC_ and total CEC respectively, with an increase in MMC can be noticed from these figures. In particular, lesser values of *S*_*a*_ and *S*_*p*_ corresponding to lower values of MMC and greater values of (Ca/Na)_CEC_ and vice versa can be visualized from these figures. A closer examination of surface profile of Fig. 6a further reveals that *S*_*a*_ becomes constant at (Ca/Na)_CEC_ of approximately 30, for the whole range of MMC. On the contrary, *S*_*p*_ attained peak value and then became constant only when the ratio of (Ca/Na)_CEC_ exceeds 30 and MMC lies above 50% (Fig. 6b). However, when MMC is less than 40%, steady vertical rise in surface profile of *S*_*p*_ with an increase in (Ca/Na)_CEC_ ratio is noticeable. On the other hand, a remarkable lower values of (Ca/Na)_CEC_ and exorbitantly higher values of MMC can be noticed when *S*_*p*_ exceeds 5000 kPa, as is seen from Fig. 6b. From the surface profile sloping towards MMC, it can be inferred that (a) *S*_*a*_ is largely governed by MMC for its whole range, not the ratio of (Ca/Na)_CEC_, and (b) *S*_*p*_ is seemed controlled by MMC only when it is above 50%.

Similarly, steady increase in *S*_*a*_ and *S*_*p*_ (diagonally upward) with an increase in total CEC and MMC can be witnessed from Fig. 7a. The observation made from figure is in well agreement with the results of Pedarla et al.^74^ and Tahasildar et al.^11^, who have reported an increment of swelling properties with MMC. As such, surface profile nearly vertical for *S*_*p*_, but leaned towards total CEC for *S*_*a*_. Inclination of surface profile towards chemical parameter may be an indication that *S*_*a*_ is more impacted by total CEC, rather than MMC. Understandably, perfect vertical rise substantiates equal influence of both total CEC and MMC of a given soil on *S*_*p*_ parameter. It can also be concluded from Fig. 7b that the combination of total CEC and MMC are best parameters to predict the swelling pressure of expansive soils.

**Figure 8:**
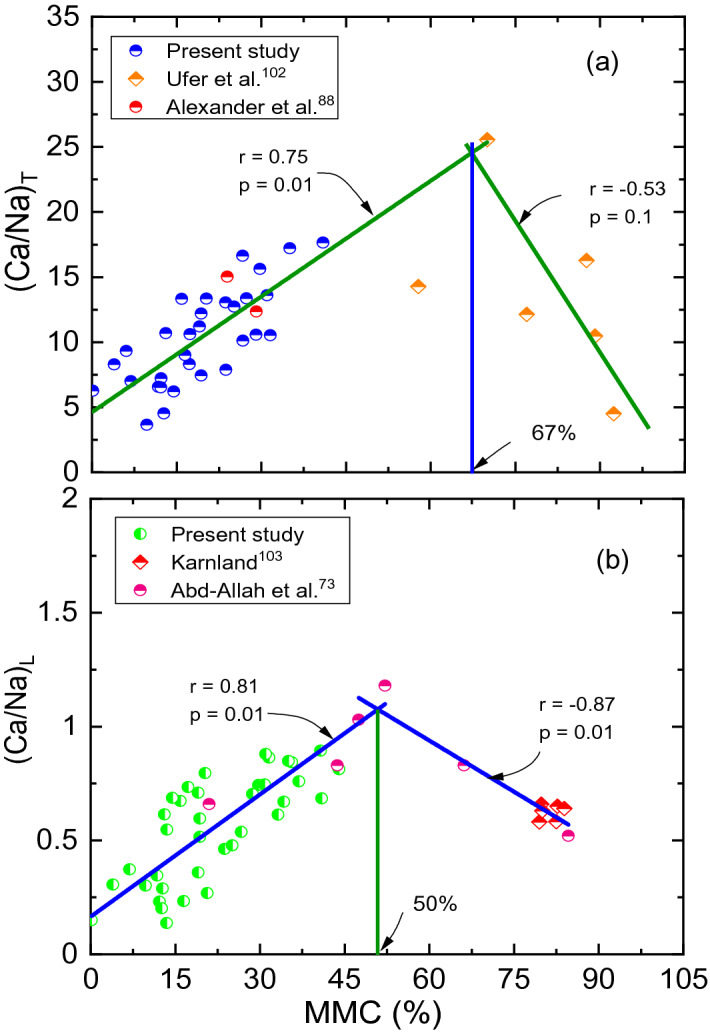
Relationship between MMC and Ca/Na Ratio of (**a**) total and (**b**) Leachable forms.

**Figure 9:**
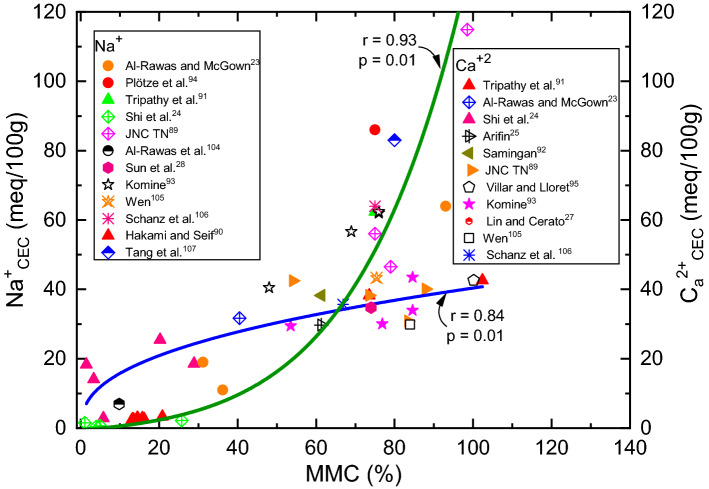
Variation of Na_CEC_ and Ca_CEC_ with MMC for expansive soils.

**Figure 10:**
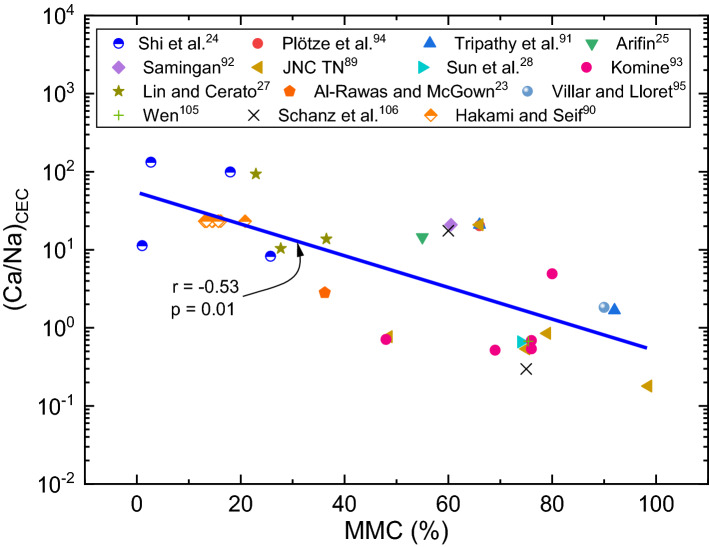
Variation of (Ca/Na)_CEC_ with MMC for expansive soils.

**Figure 11:**
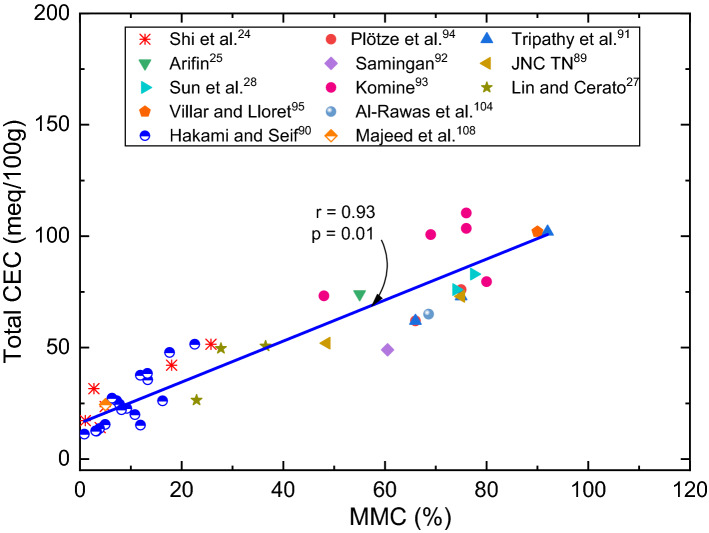
Variation of total CEC with MMC of expansive soils.

As evident from Figs. 3, 4, 5, 6 and 7, surface profiles are not similar among total, leachable, and exchangeable forms of chemical parameters, though MMC range is bracketed. This demonstrates a distinct impact of chemical parameters on *S*_*a*_ and *S*_*p*_, though the degree of disparity appears to be indifferent. Conversely, surface profiles are reasonably similar between *S*_*a*_ and *S*_*p*_ irrespective of total, leachable or exchangeable forms of chemical parameters. It portrays a fact that there is a significant variance in chemical compositions and even the availability of parameters form corresponding to a particular MMC value. In order to verify and confirm the above facts, variations of (Ca/Na)_T,_ (Ca/Na)_L,_ (Ca/Na)_CEC_, and total CEC versus MMC are plotted, as shown in Figs. 8, 9, 10 and 11. Please note that the maximum MMC quantified for the soils used in the study is 43%. Therefore, relevant data for MMC beyond this value were collected from the literature and superimposed on Figs. 8, 9, 10 and 11. It is obvious from Fig. 8 that as MMC increases, (Ca/Na)_T_ and (Ca/Na)_L_ increases, reaches a peak at MMC of 67% for total form and 50% for leachable form, and thereafter, decreases with further increment of MMC. Incidentally, this observation excellently corroborates with the inferences made with Fig. 3, wherein it is pinpointed that *S*_*a*_ and *S*_*p*_ decreases when (Ca/Na)_T_ exceeds 20. The decrease in (Ca/Na)_T_ and (Ca/Na)_L_ indicates a fact that with an increase in MMC in a given soil so does total and leachable Na content in it, which in turn highlights that MMC increases soils become rich in total and leachable Na content, not the total Ca content.

Many studies have reported significantly higher values of CEC for mineral rich soils such as bentonite (60–150 meq/100 g) vis-à-vis natural expansive soils (0–70 meq/100 g)^12,14^. Based on these disclosures, it is prudent to conclude that there should be an inherent relationship between mineral content and exchangeable capacity of cations. To verify and confirm this fact, efforts are devoted to develop relationships for Na_CEC_, Ca_CEC_, (Ca/Na)_CEC_ ratio and total CEC with MMC, as shown in Figs. 9, 10 and 11 respectively. Trends shown in these figures bear very practical significance in a sense that it is important to know the relationship between chemical and mineralogical parameter as both these parameters are key to predict volume change behaviour of expansive soils. From the above observations, it can be deduced that contents of Ca and Na, say in total, leachable or exchangeable form, are depended upon constituent minerals content. These findings well substantiate the results of Foster^32^, who stated that ionization of Na or Ca depends on the constituent minerals content.

Trends of these figures manifest a fact that individual Na_CEC_ and Ca_CEC_ displays an exponential relationship, while the ratio (Ca/Na)_CEC_ and total CEC portrays linear relationship with MMC for expansive soils. It can be observed from Figs. 9, 10 and 11 that Na_CEC_, Ca_CEC_ and total CEC increases, and (Ca/Na)_CEC_ decreases with an increase in MMC. As striking from Fig. 8, the value of Ca_CEC_ is higher in comparison with Na_CEC_ for a given MMC up to 65% and beyond this value, Na_CEC_ exhibited prominence over its counterpart ion. The same statement might be valid true from Figs. 10 and 11, but linear fitting seems masking the effect. A close review of Fig. 10 makes it noticeable that (Ca/Na)_CEC_ is clearly less than unity when MMC exceeds 65%. The decrement of (Ca/Na)_CEC_ versus MMC (Fig. 10) is quite analogous to Fig. 6 response. Incidentally, MMC of 67% is identical between the intersection point of Na_CEC_ and Ca_CEC_ (Fig. 9) and peak of (Ca/Na)_T_ (Fig. 8). These inferences further remarkably substantiate the results of Fig. 6b, wherein it is found that surface profile slopes towards MMC when it is above 50%. Similarly, the increment of total CEC with MMC is identical between Figs. 7 and 10. The authors opine that the explanation given in the context of Figs. 6 and 7 holds true here as well.

The following inferences were further made from Figs. 3, 4, 5, 6 and 7: (a) *S*_*a*_ of natural expansive soils is largely confined to 30%, while the same for bentonite soils has reached as high as 150%, (b) *S*_*p*_ of natural expansive soils is found to be less than 120 kPa, while the same for bentonite soils is found to be exorbitantly greater (i.e. above 5000 kPa), (c) MMC in natural expansive soils is mostly found below 45%, while the same in bentonite soils is reported above 90%, (d) the effect of (Ca/Na)_CEC_ ratio and total CEC are predominant on *S*_*p*_ than on *S*_*a*_, and (e) total form of Ca/Na ratio is attracted merit over leachable form in predicting *S*_*a*_ and *S*_*p*_.

From the comprehensive test results, it has been noticed that the minimal amount of *S*_*a*_ and *S*_*p*_ can be envisioned at MMC < 10%, (Ca/Na)_T_ > 15, (Ca/Na)_L_ > 1, and (Ca/Na)_CEC_ > 10 respectively. In order to constrain the swelling behaviour of expansive clays within the range that would pose no potential threat to structure, it is recommended to ensure that soils comprise of mineral and chemical compositions not exceeding the above specified limits.

## Discussion

Montmorillonite among minerals and Na and Ca contents among chemical parameters are chief constituents of expansive soils. Furthermore, Na and Ca contents are also the structural constituents of montmorillonite mineral, which is evident from its empirical formula (i.e. Na_0.2_Ca_0.1_Al_2_SiO_10_(OH)_2_(H2O)_10._ As per the Foster^32^, variances in swelling characteristics are associated with chemical parameters, degree of ionization, and type and amount of associated exchangeable cations. The results presented herein evidently prove the same fact.

Many studies report that MMC and cations concentration enhance the swelling behaviour by forming diffuse double layer upon hydration^8,33,34^. Hydration is the prime cause for swelling soils to exhibit volume change behaviour. Incidentally, both mineral (MMC) and chemical (Na and Ca) parameters prone to undergo hydration phenomenon, and thereby accentuate swelling in expansive soils^32,75^. As discussed during the explanation of Fig. 2 about triggering mechanism of swelling between chemical and mineral components, it is sensible to hypothesize that chemical effect occurs first at microscale level and the same gets manifested at macroscale level, essentially replicating as mineral behaviour^75^. Revisiting the hydration phenomenon, Na or Ca contents in soils acts as a wedge between the mineral layers, so does the swelling behaviour. It certifies that the swelling is primarily induced by the chemical parameters and followed by MMC. Generally, hydration of cations is quick and rapid in comparison with minerals^76,77^. For ex: there is a 20 fold difference in ionization between Na and Ca ions in the case of bentonite soil^32,78,79^. Similarly, Zhang et al.^80^ postulated that Na-montmorillonite hydrates more than Ca-montmorillonite does at the same water content. Differing in the ionization capabilities between Na and Ca ions can be reasoned out for the disparity in the values of swelling properties, as shown in Figs. 3, 4 and 5. The increase in ionization of either Na or Ca with an increase in MMC could be a logical hypothesis for the increase in swelling property as the MMC increases, of response depicted in Figs. 3, 4 and 5. Furthermore, the cations that exist between sheets of clay minerals generally act as bridges^76,81^. During the hydration phenomenon, these links/bridges result in the formation of thicker diffuse double layer around the clay particles. It is obvious to expect that as the MMC increases so does thickness of diffuse layer and hence, swelling in soils. Validating these statements, continues increase in swelling property with an increase in MMC can be witnessed from Figs. 3, 4 and 5. On the other hand, there is an increase and decrease of Ca/Na ratio with MMC, as apparent from Fig. 8. This trend of *S*_*a*_ and *S*_*p*_ increase and decrease with (Ca/Na)_T_ (Fig. 3) very well mimics the variation of (Ca/Na)_T_ with MMC (Fig. 8).

As is true from Fig. 9, there is a disparity in the quantity of exchangeable cations for a given MMC. It can be interpreted from trends in Fig. 9 that divalent Ca^++^ cations are dominant in expansive soils up to MMC of 65% and beyond this percentage, prevalence of monovalent Na^+^ is clear corresponding to bentonite soils. It is thus judicious to conclude from the figure that the soils with MMC below 65% can be categorized as natural expansive soils, which as per Fig. 9 are evidently dominated with Ca^++^ cations. Results presented in Fig. 6 are in perfect agreement with this fact, as it is seen that *S*_*a*_ values are appreciably low even when (Ca/Na)_CEC_ values are markedly high at MMC of negligible value. With the dominance of Ca over Na, monovalent cations will be replaced with divalent Ca^++^ ions, resulting in the condensation of thickness of diffuse double layer^32^. Moreover, the atomic radius of Na is lesser than that of Ca. This has tremendous implication as it directly impacts affinity to water absorption, which in turn affects the thickness of diffuse double layer over the clay platelets.

It is understood that *S*_*a*_ of soils is influenced by the hydration of cations content, whereas the same is not prevalent in case of *S*_*p*_ due to the applied loading condition. Under no hydration condition the effect of chemical parameters is less, which is logical to link differences in behaviour of *S*_*a*_ and *S*_*p*_, as evident from Fig. 6. Certifying this, the surface profile of *S*_*p*_ remained vertical, although (Ca/Na)_CEC_ and MMC varied. The vertical profile is due to the variance of (Ca/Na)_CEC_ with MMC of soils. From this, it is evident that there is an inherent relationship exists between exchangeable cations and MMC. The same is evident in the case of total and leachable forms as well (refer to Figs. 3, 4 and 5). Based on this understanding, it can be discerned from Fig. 6 that *S*_*a*_ and *S*_*p*_ values are significantly lower when MMC is below 40%, though wider variance in the values of (Ca/Na)_CEC_ is explicit. Conversely, more affinity to water by Na ions obviously results in greater volume change or swelling^24^, as the same is true that greater values of swelling property at higher content of MMC from Fig. 6. These statements well corroborate with the results of Liu et al.^82^, who have reported that *S*_*a*_ would be reduced when Na-montmorillonite is mixed with the solution rich in Ca ions. Reduction in ionization strength by the replacement of Na ions with Ca ions, which in turn abates the water adsorption capacity^83^, may be a cause for waning of trend with further increase of (Ca/Na)_CEC_ as shown in Fig. 6. The above findings excellently endorse the results of Foster^32^, who have stated that ionization of Na or Ca depends on the constituent mineral contents.

It is important to highlight here that the interlayer space for Ca saturated montmorillonite can be higher than Na; this however depends on the relative humidity of the environment^81^. Acknowledging this fact, Watanabe and Sato^84^ investigated the same and reported that the basal spacing of Ca saturated montmorillonite is greater than Na until relative humidity of 90%. At relative humidity of 100% (i.e. third water layer), the basal spacing found to be same (i.e. 18.8 A°)^85^. But under water saturated conditions, both Na and Ca saturated montmorillonites do not exhibit similar interlayer spacing corresponding to three water layers. It is in confirmation with the studies of Teich-McGoldrick et al.^86^, who have reported an increment in interlayer spacing with an increment in water content. This is due to the fact that Ca cations provide stronger bonding force within the interlayers, and thereby, allow lesser water molecules into these interlayers. As appraised earlier, the ionization capacity of Na is 20 folds greater than Ca^32^. This may be a reason why Na-montmorillonite is selected as a barrier material in hazardous landfills. From the ongoing discussion, it is clear that the Na and Ca saturated soils possess same interlayer space at 100% relative humidity, but not for the water condition. The above inferences validating to the present study, samples in the present study were analyzed at an ambient temperature, and at that condition, the interlayer spacing of Ca-saturated will be greater than Na as per Watanabe and Sato^84^. Whereas in the case of water saturated, which is the current tested environment, the interlayer spacing with Na is greater than Ca, so does the swelling behaviour.

It can be noted that total CEC is the sum effect of Na, Ca and other cations. As evident from Fig. 11, sum effect is found to be increased with an increase in MMC. Such analogy validates the results presented in Fig. 7, wherein it can be seen a continuous increase in *S*_*a*_ and *S*_*p*_ with an increase in MMC and total CEC. Studies also reported that, in addition to montmorillonite, the exchange phenomenon in kaolinite soil group can also exhibit minor amount of swelling upon on hydration, which might be a reason behind the scatter of data in Fig. 7. From the foregoing discussion, it can be understood that constituent different chemical elements and their quantities might vary with the mineralogy in soils. In this context, it can be stated that natural expansive soils are generally composed with different minerals with no dominance to particular mineral, so does the quantity of other cations. Also evident from the formula mentioned above, montmorillonite composes of other elements such as Al, Si, Fe and Mg. Because of their trivial quantity, these elements may not affect the swelling behaviour largely alike Na and Ca does, but surely effect the total CEC of soils. From this, it is obvious that as MMC increase, so does the other elements as well, which may be a reason behind the linearity observed in Fig. 11.

## Conclusions

In the present study, 46 numbers of different expansive type soil samples were collected from diverse locations across India. Mineralogical and chemical contents of these soil samples were analyzed in order to investigate their combined impact on swelling property. The results presented are unique and bear a practical significance for field engineers, as they can predict the swelling behaviour based on the measured chemical and mineral parameters. From the extensive experimentation and interpretation of obtained results, the following salient conclusions were made:

1. The analysis and interpretation of exhaustive results presented in 3D graphical forms and substantiated further with 2D graphs clearly demonstrate the merit and necessity of the combined impact of chemical and mineralogical parameters on swelling properties.
2. The various results vividly establish that all three forms including total, leachable and exchangeable of Ca and Na contents have had an influence and it is distinct among them on swelling properties of expansive soils.
3. The maximum value of MMC, *S*_*a*_, and *S*_*p*_ of expansive soils used in the study are measured as: 43%, 18%, and 93 kPa respectively. Further, the maximum *S*_*a*_ and *S*_*p*_ is measured at (Ca/Na)_T_ of 11.2 and (Ca/Na)_L_ of 1.1.
4. The study recommends (Ca/Na)_T_ + MMC combination for accurate measurement of *S*_*a*_ and total CEC + MMC combination for accurate measurement of *S*_*p*_ parameters.
5. It is in general noticed from the trends that (Ca/Na)_T_ and total CEC along with MMC have a non-linear and (Ca/Na)_L_ and (Ca/Na)_CEC_ along with MMC have linear correlation with *S*_*a*_ and *S*_*p*_ parameters.
6. It has been confirmed that Ca and Na contents, either in total or leachable or exchangeable form, are depended on constituent minerals content, which is in conformation with the statement of Foster^32^ that ionization of Na or Ca depends on the constituent minerals content.
7. It is evident from the study that divalent Ca^++^ cations are dominant in expansive soils up to MMC of 65% and beyond this percentage, prevalence of monovalent Na^+^ is clear.
8. From the comprehensive analysis of results, it is advocated to confine the MMC, (Ca/Na)_T_, and (Ca/Na)_L_ values to < 10%, > 15, and > 1 respectively. In order to restrict the swelling, it is advised to avoid the expansive soils with (Ca/Na)_CEC_ below 10, which will be dominated with Na content and susceptible to unpredictable volume change behaviour.
